# Chloroplast genome analysis of *Dendrocalamus × mutatus* and its implications for bamboo classification

**DOI:** 10.1186/s12870-025-07199-x

**Published:** 2025-09-01

**Authors:** Shijie Huang, Guojing Yu, Yong Wang, Huibin Gao, Chaomao Hui, Naresh Vasupalli, Xinchun Lin

**Affiliations:** 1 National Key Laboratory for Development and Utilization of Forest Food Resources, Zhejiang A & F University, Lin’an, Hangzhou, Zhejiang 311300 China; 2 Key Laboratory of Bamboo Science and Technology of Ministry of Education, Bamboo Industry Institute, Zhejiang A & F University, Lin’an, Zhejiang 311300 China; 3 College of Arts and Design, Zhejiang A & F University, Lin’an, Zhejiang 311300 China; 4 Forest and Bamboo Resources Conservation and Cultivation Institute, Yibin Forestry and Bamboo Industry Research Institute, Yibin, Sichuan China; 5 Southwest Forestry University, Kunming, Yunnan China

**Keywords:** *Dendrocalamus × mutatus*, Chloroplast genome, Phylogenetic analysis, Bamboo species, Genomic comparison

## Abstract

**Background:**

The bamboo species *Dendrocalamus × mutatus* T.P.Yi & B.X.Li (*D. mutatus*) holds great economic and ecological importance in China. Although previously *D. mutatus* was presumed to be a hybrid of *Bambusa grandis* and *Bambusa pervariabilis*, its taxonomic status has remained uncertain. Therefore, we combined plastomes and nuclear SSR datasets, in addition to morphological data, to refine our understanding of the taxonomic status of *D. mutatus*.

**Results:**

The chloroplast genome of *D. mutatus* exhibits a typical quadripartite structure, comprising a large single-copy region (LSC), a small single-copy region (SSC), and two inverted repeat regions (IRa and IRb), containing a total length of 139,432 bp. Comparative genomic analyses revealed extremely high similarity between *D. mutatus* and *D. yunnanicus*, differing by only two single-nucleotide polymorphisms (SNPs). In contrast, a greater divergence was observed when compared with *D. sikkimensis* (six SNPs and one four-base insertion). Phylogenetic reconstruction using the Maximum Likelihood and Bayesian Inference method based on chloroplast genomes strongly supported the close relationship between *D. mutatus* and *D. yunnanicus*, while distinctly separating them from the previously proposed parent species *Bambusa grandis* and *Bambusa pervariabilis*. Morphological comparisons further confirmed the similarity between *D. mutatus* and *D. yunnanicus*, particularly with respect to the absence of conspicuous culm sheath auricles and oral setae, as well as the lack of fimbriate hairs on the ligule. Nuclear SSR marker analyses also showed identical predominant allele at the SSR 23 and 24 loci between *D. mutatus* and *D. yunnanicus*. Collectively, the comprehensive integration of chloroplast genome data, nuclear SSR evidence, and morphological observations supports the conclusion that *D. mutatus* is a synonym of *D. yunnanicus* rather than a hybrid of *Bambusa grandis* and *Bambusa pervariabilis*.

**Conclusions:**

This research provides a comprehensive understanding of the chloroplast genome of *D. mutatus*, providing valuable insights that enhance the taxonomic resolution and conservation strategies for bamboo species.

**Supplementary Information:**

The online version contains supplementary material available at 10.1186/s12870-025-07199-x.

## Background

Bambusoideae subfamily is the third largest in the grass family (Poaceae). Within this subfamily, woody bamboos are distinguished by their woody culms and unique flowering cycles exhibiting long intervals from 40 to 120 years [1]. They are widely distributed in temperate and tropical regions, especially in some countries, e.g. China, India, and Japan. Bamboos are highly valued for their economic and ecological importance, being widely used in furniture, musical instruments, construction, and papermaking [2–5]. Bamboo has a high economic value and is widely used in the preparation of furniture, musical instruments, building materials and papermaking [6–11]. Traditionally, bamboo species are classified based on morphological features such as culm structure, leaf shape, branching patterns, and flowering traits [12, 13]. However, species identification is very difficult when based only on morphology, because many species share highly similar vegetative features and reproductive materials are often lacking [14–18].

Therefore, molecular approaches have become essential for accurately resolving the relationships and identification of bamboo species. The chloroplast genome has emerged as an effective tool for addressing these classification challenges due to its simple structure, stability, low nucleotide substitution rate, and conserved gene order [19]. Most plant chloroplast genomes have conserved quadripartite structures, including a large single copy (LSC) region and a small single copy (SSC) region separated by a pair of inverted repeats (IRs). The chloroplast genome generally contains 110–140 genes that encode tRNAs, rRNAs, photosynthesis-related proteins, and other proteins involved in chloroplast function [20–22]. The chloroplast genome has a lower nucleotide substitution rate than the nuclear genome and exhibits highly conserved gene order and gene content across most plant species, making it ideal for evolutionary and comparative studies [19, 23–25]. Consequently, coding and non-coding chloroplast DNA sequences or sometimes complete chloroplast genome sequences have been widely used for phylogenetic studies of higher plants [26–28]. Recent advances in cost-effective next-generation sequencing techniques have greatly facilitated the development of numerous chloroplast genomes extending gene-based phylogenetics to broader phylogenomics [29]. In particular, chloroplast genomes have been successfully utilized in the classification and evolutionary studies of bamboo species, effectively addressing the complex challenges associated with conventional classification [12, 30–37].

The genus *Dendrocalamus* comprises 66 species, primarily distributed across China, the Indian subcontinent and southeast Asia. Among the 66 *Dendrocalamus* species, 30 occur in southwestern China [38–40]. Among them, *Dendrocalamus × mutatus* T.P.Yi & B.X.Li (*D. mutatus*) [41], is predominantly cultivated in Sichuan Province, China. This species is highly valuable for its use in landscape and raw material for pulp and paper production [42]. This species was previously identified as a hybrid between *Bambusa grandis* (syn. *Dendrocalamopsis daii*) and *B. pervariabilis* based on morphological observations, but its exact taxonomic status remains controversial [41]. However, we observe that *D. mutatus* and *D. yunnanicus* have similar morphological characteristics, implying that they could be conspecific. The morphological similarity between *D. mutatus* and *D. yunnanicus* makes it challenging to distinguish them solely on morphological, highlighting the need for further evidence, such as molecular data. Therefore, our study aims to clear the taxonomic identity of *D. mutatus* based on chloroplast genome evidence. Therefore, our study aims to clarify the taxonomic identity of *D. mutatus*, specifically to determine whether it is a hybrid or should be considered synonymous with *D. yunnanicus*, based on genome evidence.

## Result and discussion

### Characteristics of the *D. mutatus* chloroplast genome

**Fig. 1:**
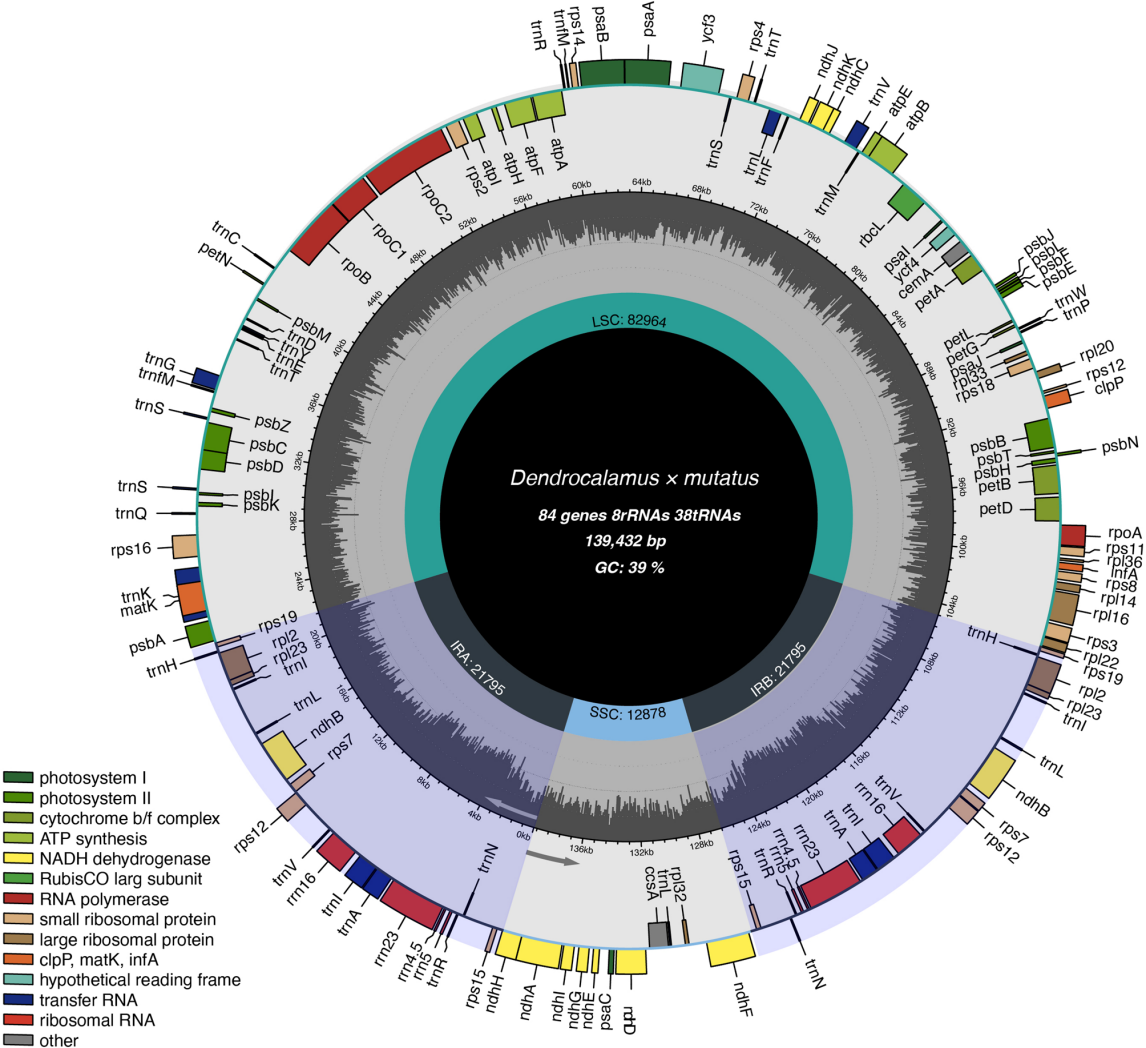
The circular chloroplast genome map of the *D. mutatus*. Genes represented on the outside and inside the map are transcribed clockwise and anticlockwise, respectively. Genes belonging to same functional groups are mentioned in same colour. The inner circle in the grey colour corresponds to GC content. The SSC region, LSC region are mentioned in cyan and blue colour regions respectively. Inverted repeats (IRa and IRb) are indicated in gray colour

We performed chloroplast whole-genome sequencing of *D. mutatus*, which produced an initial dataset of 3.25 Gb containing 10,832,384 raw reads. Following thorough quality assessment and trimming procedures, the refined dataset comprised 3.22 Gb containing 10,720,626 high-quality reads, with individual reads averaging 150 bp. We have the complete chloroplast genome of *D. mutatus* by mapping the quality reads to the reference genome *D. latiflorus* (NCBI ID: NC_013088.1). A total of 10,610,203 (98.97%) quality reads were mapped to the reference genome and assembly analysis revealed a circular genomic structure measuring 139,432 bp in length containing a GC content of 38.92% (GenBank accession number: PQ369414.1). The *D. mutatus* chloroplast genome generated is a typical quadripartite structure, consisting of a large single-copy region (LSC) of 82,964 bp (59.50%), a small single-copy region (SSC) of 12,878 bp (9.24%), and a pair of inverted repeat regions (IRa and IRb) measuring 21,795 bp (15.62%) each (Table S1, Fig. 1). The nucleotide composition was 31% adenine (A), 19% thymine (T), 20% cytosine (C), and 30% guanine (G). The GC content varied across regions, being 33.7% in the SSC, 37.0% in the LSC, and 44.2% in both IRa and IRb (Table S1). These results such as the composition and proportions of the SSC, LSC, and IR regions are similar to those within the genus *Dendrocalamus*, such as *D. sikkimensis*, *D. latiflorus*, *D. farinosus*, and *D. sinicus* [30, 43–45]. This observation indicates a high degree of conservation in the chloroplast genome.

Using CPGAVAS2 for genome annotation, we identified a total of 130 genes in the *D. mutatus* chloroplast genome, including 84 protein-coding genes (PCGs), 8 ribosomal RNA (rRNA) genes, and 38 transfer RNA (tRNA) genes (Fig. 1; Table 1). Among the genes, 19 genes contain introns: 18 of these have two exons and one intron each, while the *ycf3* gene has three exons and two introns. These intron-bearing genes are distributed as follows: ten are located in the LSC region, eight are present in both IR regions, and one is found in the SSC region (Table 2). The longest and shortest introns are present in tRNAs, namely *trnK-UUU* with 2,505 bp, and *trnL-UAA* with 539 bp, respectively. The t*rnK-UUU* gene also has the longest intron in the chloroplast genome of other bamboo species, such as *D. farinosus*,* D. sikkimensis* and *D. latiflorus* [30, 43, 44]. Similar to our results, the shortest intron 539 bp *trnL-UAA* is the shortest intron in *D. sikkimensis*. However, in contrast to our results, *D. farinosus* and *D. latiflorus* have *rps12* with a shortest intron of 537 bp [30, 44].

**Table 1:** Genes present in the chloroplast genome of *D. mutatus*

Category	Gene group	Gene name
Photosynthesis	Subunits of photosystem I	*psaA*,* psaB*,* psaC*,* psaI*,* psaJ*
	Subunits of photosystem II	*psbA*,* psbB*,* psbC*,* psbD*,* psbE*,* psbF*,* psbH*,* psbI*, *psbJ*,* psbK*,* psbL*,* psbM*,* psbN*,* psbT*,* psbZ*
	Subunits of NADH dehydrogenase	ndhA*, ndhB*(2),*ndhC*,* ndhD*,* ndhE*,* ndhF*, *ndhG*,* ndhH*,* ndhI*,* ndhJ*,* ndhK*
	Subunits of cytochrome b/f complex	*petA*, petB*,petD*,*petG*,* petL*,* petN*
	Subunits of ATP synthase	*atpA*,* atpB*,* atpE*, atpF*,*atpH*,* atpI*
	Large subunit of rubisco	*rbcL*
Self-replication	Proteins of large ribosomal subunit	*rpl14*, rpl16*, rpl2*(2),* rpl20*,* rpl22*,* rpl23(2)*, *rpl32*,* rpl33*,* rpl36*
	Proteins of small ribosomal subunit	*rps11*, rps12**(2),* rps14*,* rps15(2)*, rps16*, *rps18*,* rps19(2)*,* rps2*,* rps3*,* rps4*,* rps7(2)*,* rps8*
	Subunits of RNA polymerase	*rpoA*,* rpoB*,* rpoC1*,* rpoC2*
	Ribosomal RNAs	*rrn16S(2)*,* rrn23S(2)*,* rrn4.5 S(2)*,* rrn5S(2)*
	Transfer RNAs	trnA-UGC*(2),* trnC-GCA*,* trnD-GUC*,* trnE-UUC*, *trnF-GAA*, trnG-UCC*,* trnH-GUG(2)*,* trnI-CAU(2)*, trnI-GAU*(2), trnK-UUU*,* trnL-CAA(2)*, trnL-UAA*,* trnL-UAG*,* trnM-CAU*,* trnN-GUU(2)*, *trnP-UGG*,* trnQ-UUG*,* trnR-ACG(2)*,* trnR-UCU*, *trnS-GCU*,* trnS-GGA*,* trnS-UGA*,* trnT-GGU*, *trnT-UGU*,* trnV-GAC(2)*, trnV-UAC*,* trnW-CCA*, *trnY-GUA*,* trnfM-CAU(2)*
Other genes	Maturase	*matK*
	Protease	*clpP*
	Envelope membrane protein	*cemA*
	c-type cytochrome synthesis gene	*ccsA*
	Translation initiation factor	*infA*
	Component of TIC complex	ycf3**,* ycf4*

Gene*:Gene with one introns; Gene**:Gene with two introns; Gene(2):Number of copies of multi-copy genes

**Table 2:** The introns in the genes of the *D. mutatus* chloroplast genome

Gene	Strand	Location	ExonI	IntronI	ExonII	IntronII	ExonIII
*trnK-UUU*	-	LSC	38	2505	34		
*rps16*	-	LSC	40	846	218		
*trnG-UCC*	-	LSC	23	673	48		
*atpF*	+	LSC	160	837	407		
*ycf3*	-	LSC	131	732	229	724	159
*trnL-UAA*	+	LSC	35	539	50		
*trnV-UAC*	-	LSC	39	596	37		
*petB*	+	LSC	6	826	642		
*petD*	+	LSC	8	748	475		
*rpl16*	-	LSC	9	1092	408		
*rpl2*	-	IRA	409	660	428		
*ndhB*	-	IRA	775	712	758		
*trnI-GAU*	+	IRA	42	946	35		
*trnA-UGC*	+	IRA	38	811	35		
*ndhA*	-	SSC	550	1026	539		
*trnA-UGC*	-	IRB	38	811	35		
*trnI-GAU*	-	IRB	42	946	35		
*ndhB*	+	IRB	775	712	758		
*rpl2*	+	IRB	409	660	428		

**Fig. 2:**
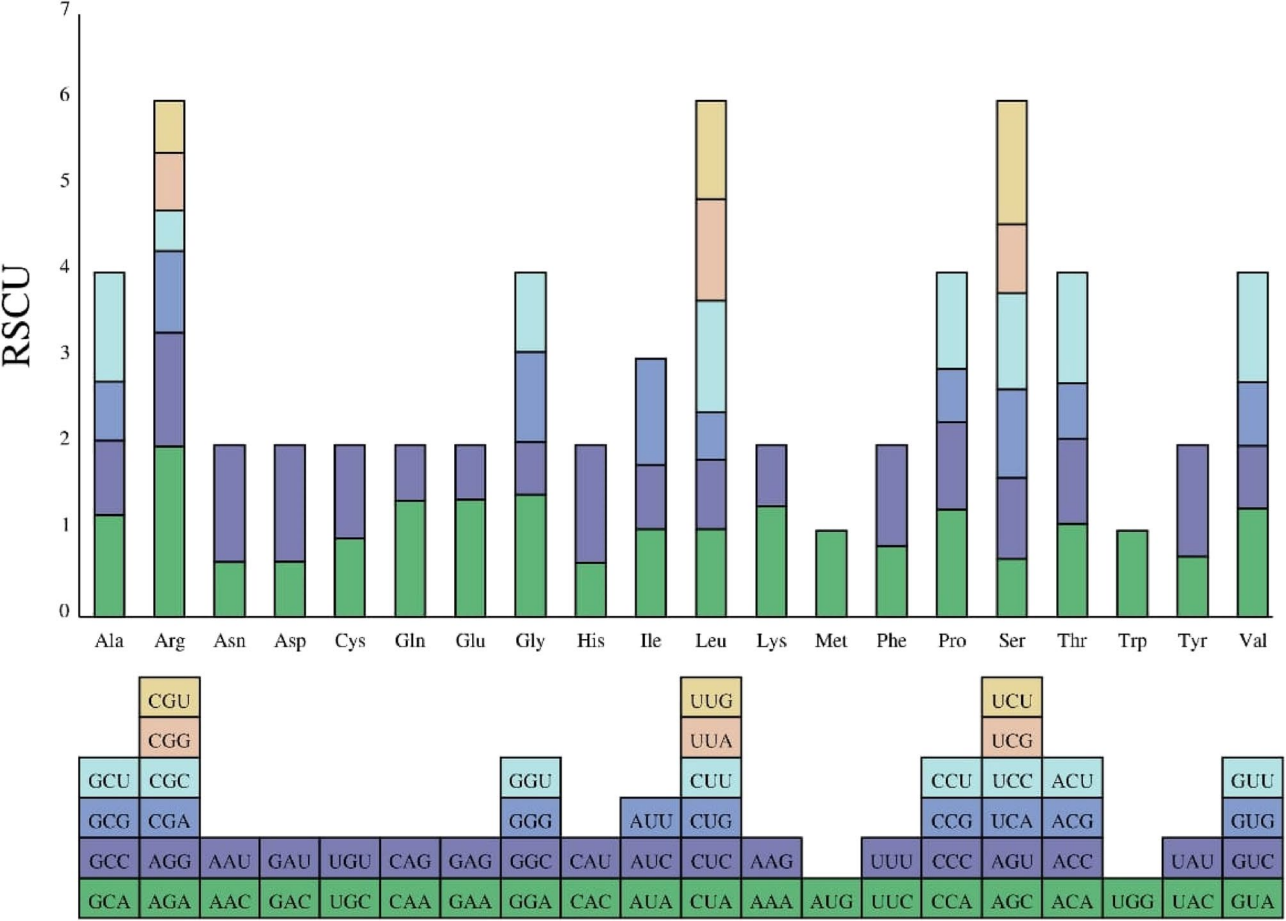
Pictorial representation of relative synonymous codon usage. Different proportions correspond to different RSCUs

Codon usage bias is thought to result from a relative balance within the cell over a long period of evolutionary selection [46, 47]. A relative synonymous codon usage (RSCU) value greater than 1 was considered to indicate the beneficial effect of amino acids. There were 28 codons for which the RSCU > 1 (Fig. 2). The RSCU values of the start codons AUG, Arginine (CGA) and tryptophan (UGG) were one. The remaining plastid protein-coding genes contain RSCU value < 1 (Fig. 2). Arginine (Arg) had the highest preference for AGA codons, with an RSCU value of 1.93 (Fig. 2). The third nucleotide in a codon showed preferential usage of A/U nucleotides over C/G, aligning with documented patterns that emerge from natural selection and mutational forces [48–50].

### Analysis of long repeats and simple sequence repeats (SSRs)

Repetitive sequences are widespread in the chloroplast genomes of terrestrial plants and present substantial polymorphism [51–53]. In the chloroplast genome of *D. mutatus*, 49 pairs of long repeats were identified, with lengths between 19 and 58 bp. These included 25 forward repeats, 6 reverse repeats, 2 complement repeats, and 16 palindromic repeats (Table S2). The majority of these repeats (75.50%) were between 20 and 30 bp long, with 10.2% exceeding 30 bp and 14.29% shorter than 20 bp. The number and length of long repeats in the *D. mutatus* chloroplast genome are comparable to those reported in other bamboo species, such as *Phyllostachys edulis*, *Sinosasa longiligulata*, *Ferrocalamus rimosivaginus*, *Gelidocalamus*, and *Indocalamus* species, which contain 34–59 forward repeats [54].

Simple Sequence Repeats are used as molecular markers in genetics to analyze genetic diversity, and population structure, and assist in breeding programs due to their high variability and widespread distribution in genomes [55–57]. A total of 28 simple sequence repeats (SSRs) were identified, all of which are mononucleotide repeats predominantly composed of adenine (A) and thymine (T). Among these, poly-A sequences exhibit four length variants ranging from 11 to 14 bp, while poly-T sequences have three length variants ranging from 11 to 13 bp (Table 3).

**Table 3:** Distribution of SSRs in the *D. mutatus* Chloroplast genome

SSR Type	Unit	Length	Number	Position on Genome (bp)
P1	A	11	5	29,391–29401,33971–33981,52983–52993,57215–57225,60687–60,697
	A	12	5	21,726–21737,57254–57265,68600–68611,71580–71591,128925–128,936
	A	13	2	67,629–67641,131964–131,976
	A	14	2	70,246–70259,95825–95,838
	T	11	8	59,891–59901,65307–65317,70153–70163,80020–80030,88839–88849,100626–100636,100644–100654,104192–104,202
	T	12	5	30,345–30356,55908–55919,92457–92468,101235–101246,104717–104,728
	T	13	1	73,443–73,455

The mononucleotide repeats composed of A and T are especially susceptible to slippage during DNA replication, which can lead to high levels of polymorphism, making them valuable tools for assessing genetic variation across populations [58]. The variability of SSRs, in particular, makes them effective markers for genetic studies, and their distribution across different chloroplast genomes provides insights into the evolutionary dynamics of plant species [32, 33, 59]. Further studies on these sequences could shed light on their role in adaptation and speciation in plants, as well as their potential applications in breeding programs and conservation genetics.

### Comparative analysis of genome structure

**Fig. 3:**
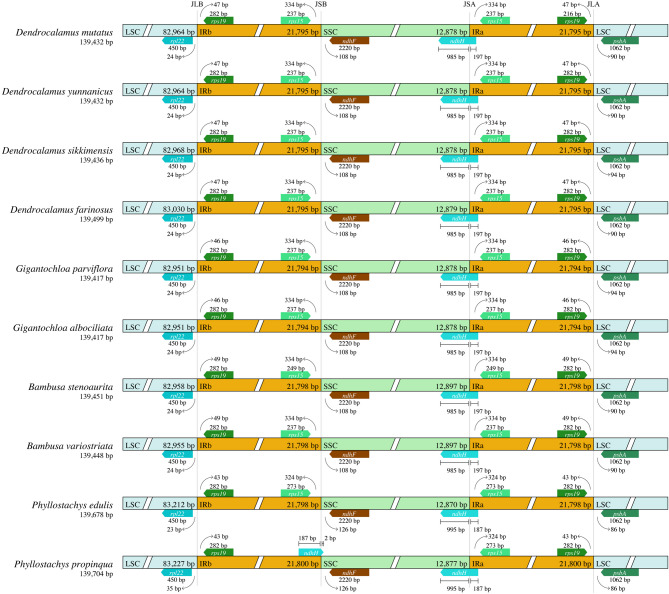
Comparison SSC, LSC, IRa and IRb border regions among ten chloroplast genomes. JLB: junction of the LSC and the IRb. JSB: junction of the SSC and the IRb. JSA: junction of the SSC and the IRa. JLA: junction of the LSC and the IRa. Genes are mentioned in colored boxes. The gaps between the genes and boundaries are mentioned in number of bps

The structure and size of plant chloroplast genomes are generally conserved [19, 25, 60]. However, expansions and contractions in chloroplast genomes occur as a result of minor changes in the size and position of the boundaries between single-copy regions and inverted repeat regions [61, 62]. These boundary shifts are often regarded as a key factor driving variation in plant chloroplast genomes [63]. In the current study, the boundaries of the IR-LSC and the IR-SSC of ten bamboo species (four genera) were compared and analyzed using the genes present at the boundaries such as *rpl22*, *rps19*, *rps15*, *ndhF*, *ndhH* and *psbA* (Fig. 3). The ten chloroplast genomes ranged from 139,417 bp (*G. nigrociliata*) to 139,704 bp (*Phyllostachys propinqua*). The *ndhH* gene is located at the SSC-IRa (JSA) boundary in all ten species across four genera, extending 187 to 197 bp into the IRa region. Additionally, the *ndhH* gene is also found in *D. farinosus* and *P. propinqua*, at the SSC-IRb (JSB) boundary. The *rpl22* gene is located in the LSC region of LSC-IRb (JLB) junction and contracted inwards by 23–35 bp from the border. The *rps19* is in both IRa and IRb regions at the junctions of IRa-LSC (JLA) and JLB and contracted inwards by 43–49 bp from the junction. The *rps19* gene expansion and contraction vary between genera while remaining consistent within the same genus. Similarly, the *rps15* gene is also present in both IRa and IRb regions at the junctions of IRb-SSC (JSB) and SSC-IRa (JSA) and contracted inwards by 324–334 bp from the border. The *ndhF* gene which is present in the SSC region at the junction of JSB, contracted 108–126 bp. Moreover, we also found that the expansion and contraction patterns in the four *Dendrocalamus* species were highly consistent, with only slight variations observed between different genera.

**Fig. 4:**
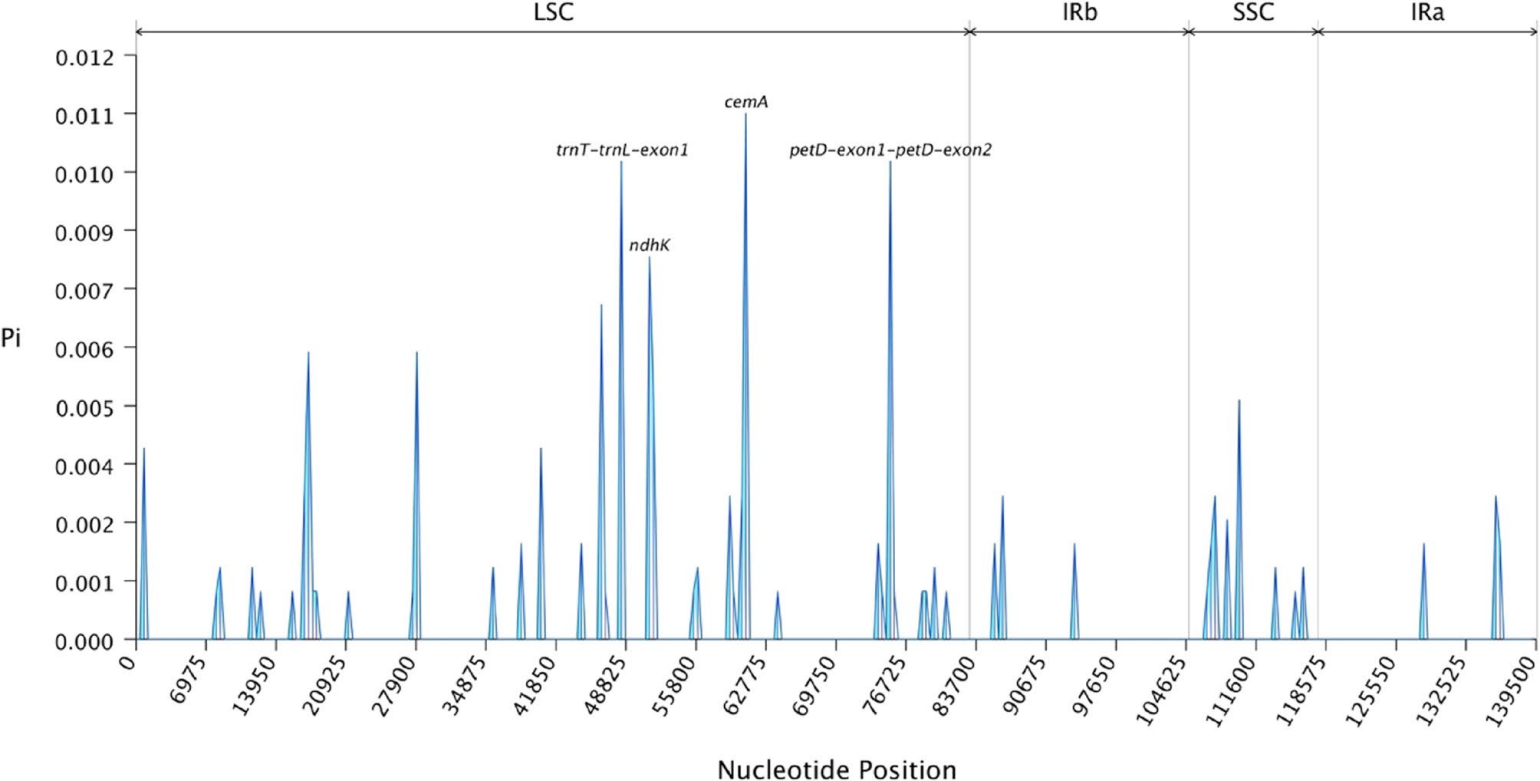
Nucleotide diversity (π) across the complete chloroplast genomes of *Dendrocalamus* species

To investigate the natural selection or nucleotide conservation in the genes of chloroplast genome, nucleotide diversity (π) patterns were analyzed in five *Dendrocalamus* species (*D. farinosus*, *D. yunnanicus*,* D. latiflorus*,* D. mutatus*, and *D. sikkimensis*) (Fig. 4). The results revealed substantial interspecific variation in the *ndhK*, *cemA*, and *petD* genes, suggesting that these loci are highly variable among the studied species. In contrast, the π values in the inverted repeat (IR) regions are relatively low, indicating a higher degree of nucleotide conservation. Previous studies have found that certain chloroplast genes, such as *petD*, which is involved in the electron transport chain of photosynthesis, exhibit higher levels of variation, reflecting potential adaptive changes to environmental conditions [64, 65].

### Phylogenetic analysis

**Fig. 5:**
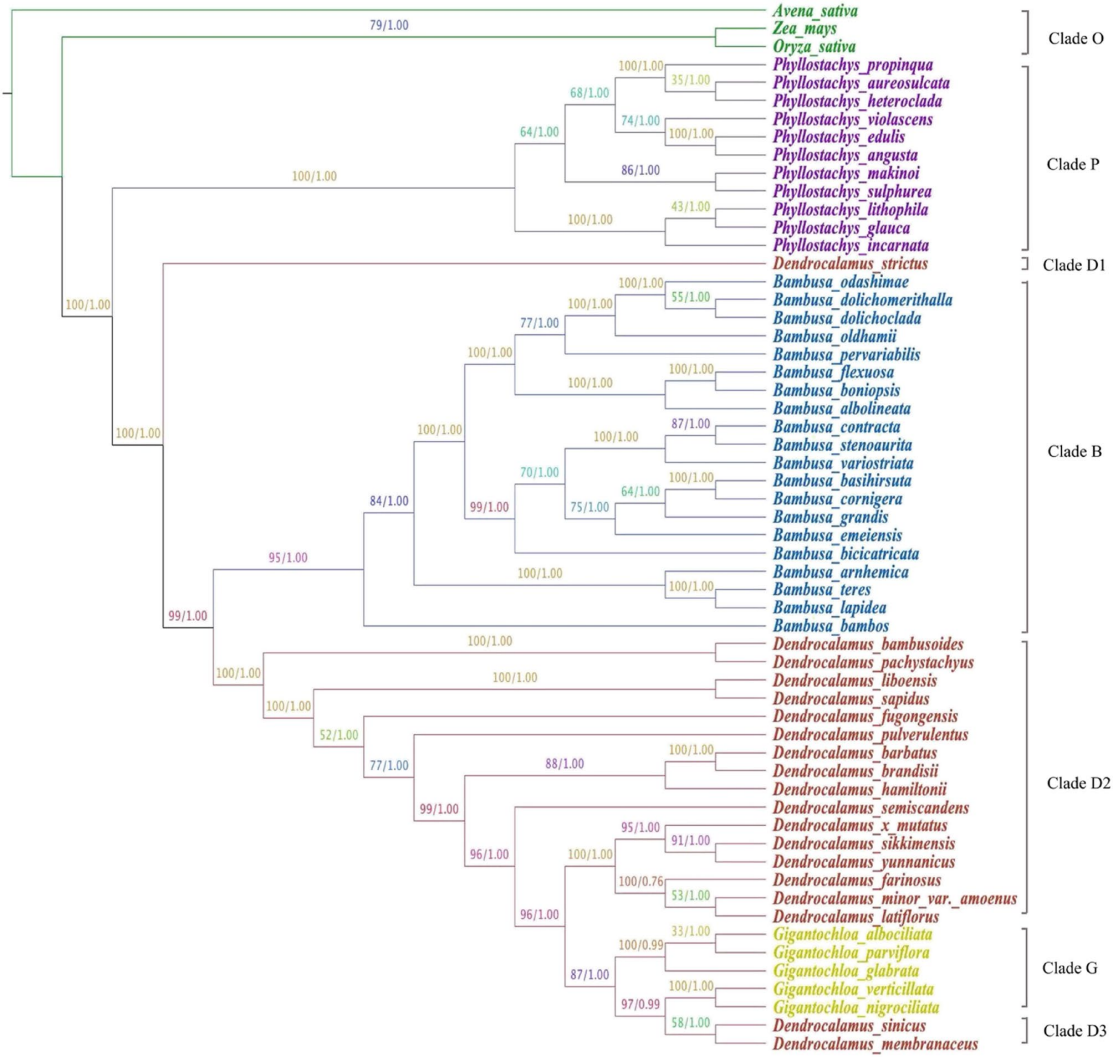
The phylogenetic relationship among 58 complete chloroplast genomes of Poaceae based on Maximum Likelihood and Bayesian analysis. Numbers above branches indicate maximum likelihood bootstrap support (BS) and Bayesian posterior probabilities (PP), respectively

Phylogenetic analysis was carried out based on an alignment of 58 Poaceae chloroplast genomes to understand the evolutionary relationship (Fig. 5, Table S3). The phylogenetic tree is divided into seven clades Clade O, Clade P, Clade D1, Clade B, Clade D2, Clade G, and Clade D3. The results showed that *Dendrocalamus* is more closely related to *Gigantochloa* than *Phyllostachys* and *Bambusa*. The phylogenetic analysis indicates *D. mutatus* is closely related to the clade comprising *D. sikkimensis* and *D. yunnanicus*. However, the proposed parents *B. grandis* and *B. pervariabilis* fall under a separate clade, Clade B. This indicates that *D. mutatus* is not a hybrid of *B. grandis* and *B. pervariabilis* but is more likely a synonym of *D. yunnanicus* or *D. sikkimensis*. Furthermore, our phylogenetic analysis reveals that the genus *Dendrocalamus* is a paraphyletic group, as species of *Bambusa and Gigantochloa* are nested within the *Dendrocalamus* clades. This observation is consistent with earlier molecular studies, which have shown that *Dendrocalamus* is paraphyletic, while *Gigantochloa* forms a monophyletic group [43]. Other studies based on nuclear and plastid markers have also suggested the paraphyly of *Dendrocalamus*, and some even indicate polyphyly, revealing a greater level of genetic divergence [66, 67]. Altogether, these findings and our results highlight a consistent pattern of non-monophyly within *Dendrocalamus*, reinforcing the need for taxonomic revision and clearer delimitation among closely related bamboo genera.

### Identification of the relationships among *D. mutatus*, *D. yunnanicus*, *D. sikkimensis*, *B. grandis*,*B. pervariabilis* and *B. pervariabilis × B. grandis*

**Fig. 6:**
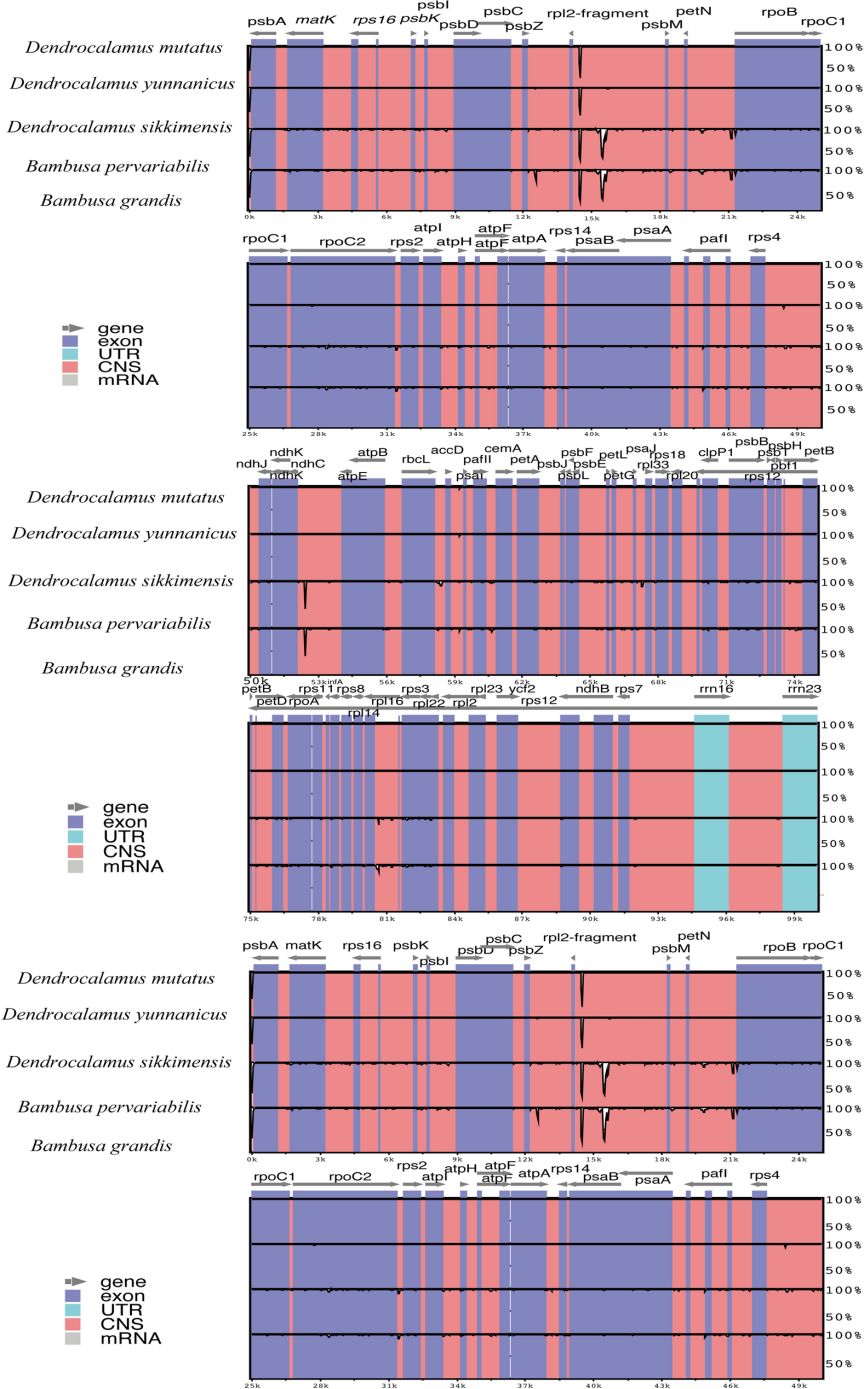
Sequence alignment of the four chloroplast genomes by mVISTA, using *D. mutatus* as the reference. The four genomes are *D. mutatus*, *D. yunnanicus*, *D. sikkimensis*, *B. grandis* and *B. pervariabilis*. The horizontal axis display the coordinates within the chloroplast genome. Different genome regions are color-coded: genes are shown in blue; conserved non-coding sequences are highlighted in dark pink; forward and backward arrows indicate the gene direction

In our study, we found that *D. sikkimensis*, *D. mutatus*, and *D. yunnanicus* are clustered together in the phylogenetic tree, indicating overall similarity in their chloroplast genomes. To identify the chloroplast genome similarity with the parental genome, a comparative analysis of five chloroplast genome sequences (*D. mutatus*, *D. yunnanicus*, *D. sikkimensis*, *B. grandis* and *B. pervariabilis*) was conducted, using *D. mutatus* as the reference genome for alignment (Fig. 6). The analysis revealed that *D. mutatus* had only two SNPs in the non-coding region in comparison to *D. yunnanicus*, while showing greater divergence from its presumed parental species, *B. grandis* and *B. pervariabilis*. Additionally, *D. sikkimensis* differed from *D. mutatus* by six SNPs and had an insertion of four bases.

**Fig. 7:**
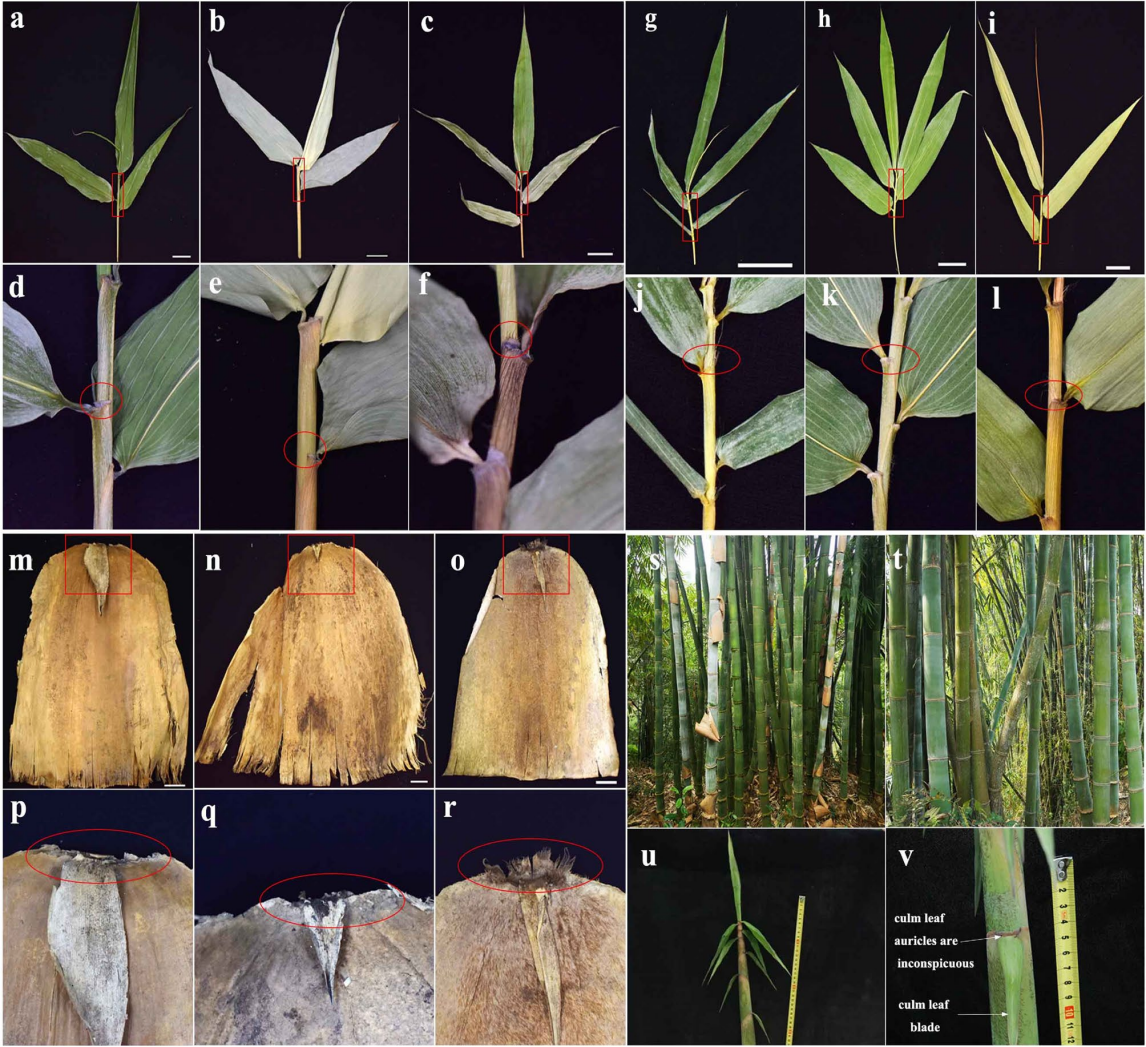
This figure shows the morphological comparison among six bamboo taxa: *D. mutatus*, *D. yunnanicus*, *D. sikkimensis*, *B. pervariabilis*, *B. grandis*, and the hybrid *B. pervariabilis × B. grandis* (a–c, g–i): Leaf blade and sheath characteristics of *D. mutatus* (**a**,), *D. yunnanicus* (**b**,), *D. sikkimensis* (**c**), *B. pervariabilis* (**g**), *B. grandis* (**h**), and *B. pervariabilis × B. grandis* (**i**). (**d**–**f**, **j**–**l**): Enlarged views of the sheath auricle and ligule regions (red circles), corresponding to a–c, g–i. (m–o): Culm sheath morphology of *D. mutatus* (**m**), *D. yunnanicus* (**n**), and *D. sikkimensis* (**o**). **p**–**r** Details of the auricle region on the culm sheath in m–o (red circles). **s**, **t** Field view of *D. mutatus* and *D. yunnanicus*, showing the clump habits and culm types. **u**, **v**: Morphological details of *D. mutatus*. (u) shows new bamboo shoot of *D. mutatus*; (v) part of young shoots, with the culm leaf blade and auricles, indicating the inconspicuous of culm leaf auricles. Scale bars: 3 cm

**Fig. 8:**
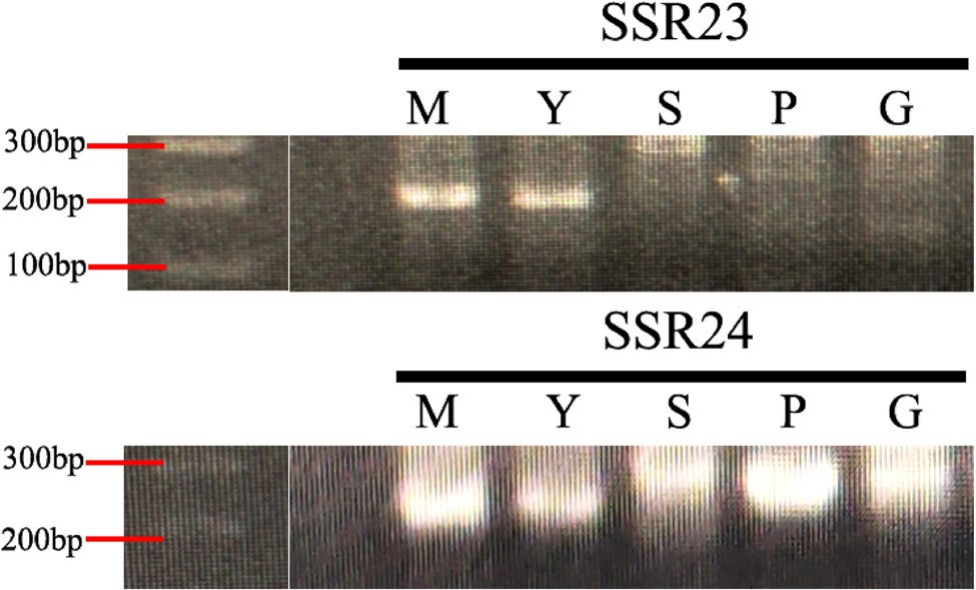
SSR polymorphism among five bamboo species. PCR was performed with SSR23 and SSR24 markers. M, *D. mutatus*; Y, *D. yunnanicus*; S, *D. sikkimensis*; P, *B. pervariabilis*; G, *B. grandis*

To complement these findings, we conducted a detailed morphological comparison among *D. mutatus*, *D. yunnanicus*, *D. sikkimensis*, *B. pervariabilis*, *B. grandis*, and their hybrid (*Bambusa pervariabilis × Bambusa grandis*), as shown in Fig. 7. We found that the morphological traits of *D. mutatus* and *D. yunnanicus* were almost indistinguishable, particularly regarding the shape and size of the culm sheath auricle, the characteristics of the oral setae and fimbriae on the ligule [41, 68]. Specifically, *D. mutatus* and *D. yunnanicus* both display small culm sheath auricle without oral setae, whereas *D. sikkimensis* has prominent culm sheath auricle with numerous, yellowish-brown bristle-like oral setae (Fig. 7, m–r). Notably, fimbriate hairs on the ligule are absent in both *D. mutatus* and *D. yunnanicus*, but are present in *D. sikkimensis*. Comparative morphological analysis further revealed that the ligule of *B. pervariabilis* is characterized by the presence of fimbriate hairs, whereas *B. grandis* lacks such hairs. In the interspecific hybrid (*B. pervariabilis × B. grandis*.), the ligule displays fimbriate hairs, resembling the condition observed in *B. pervariabilis* (Fig. 7, a–l). These subtle yet stable differences support the conclusion that *D. mutatus* and *D. yunnanicus* are conspecific, while *D. sikkimensis* is morphologically distinct. Field observations (Fig. 7, s–v) further confirmed that these two species are characterized by same clump habits and culm types. In key genes that distinguish species, such as *rpoC2*, *D. mutatus* and *D. yunnanicus* were identical, whereas obvious differences were observed compared to *B. grandis* and *B. pervariabilis* [30]. To verify these results, we collected fresh leaf samples of *D. yunnanicus* from Yunnan province, China, and sequenced the divergent regions. The sequence results were also similar to the chloroplast genome sequence indicating that the differences between the chloroplast genome of *D. mutatus* and *D. yunnanicus* were at the individual level and that their chloroplast genomes were identical (Table S4). Therefore, based on chloroplast genome analysis, we consider that *D. mutatus* not a hybrid of *B. grandis* and *B. pervariabilis*, as suggested by Professor Yi, but is a synonym of *D. yunnanicus*. However, as our conclusion is based only on plastid evidence, further studies using nuclear genomic data are necessary to confirm this taxonomic relationship. To further investigate the genetic relationships among these taxa, we performed nuclear SSR polymorphism analysis using the SSR23 and SSR24 markers (Fig. 8). The primer sequences used for identification were obtained from our previous study [34]. Out of 64 primer pairs, two were selected for further analysis (Table S5). The results showed that *D. mutatus* and *D. yunnanicus* displayed identical SSR amplification patterns at both loci, indicating no detectable variation between them at these nuclear markers. In contrast, *D. sikkimensis*, *B. pervariabilis*, and *B. grandis* exhibited distinct SSR banding profiles compared to *D. mutatus* and *D. yunnanicus*.

Overall, the congruence of plastid, nuclear, and morphological data strongly suggests that *D. mutatus* should be considered a synonym of *D. yunnanicus*. Nevertheless, further studies utilizing additional nuclear loci and more comprehensive sampling are warranted to confirm these findings.

## Conclusions

This study provides a detailed characterization of the chloroplast genome of *D. mutatus*, a bamboo species with substantial economic and ecological importance. The chloroplast genome of *D. mutatus* displays a typical quadripartite structure comprising a large single-copy (LSC) region, a small single-copy (SSC) region, and two inverted repeat regions (IRa and IRb), totaling 139,432 bp. Phylogenetic analyses based on complete chloroplast genome sequences clearly demonstrate that *D. mutatus* is closely related to *D. yunnanicus* and *D. sikkimensis*, forming a distinct clade separate from the previously proposed parental species, *B. grandis* and *B. pervariabilis*.

Comparative genomic analyses indicated extremely minimal genetic variation between *D. mutatus* and *D. yunnanicus*, differing by only two single-nucleotide polymorphisms (SNPs), while showing slightly more divergence from D. *sikkimensis*. Further, detailed morphological comparisons revealed that *D. mutatus* and *D. yunnanicus* share nearly identical traits, particularly in critical morphological features such as the absence of conspicuous culm sheath auricles, oral setae, and fimbriate hairs on the ligule. In contrast, great morphological differences were identified between these species and *D. sikkimensis*, as well as the originally proposed parental species *B. grandis* and *B. pervariabilis*. Additionally, nuclear SSR marker analyses showed identical amplification patterns for *D. mutatus* and *D. yunnanicus*, further supporting their genetic and morphological congruence. Integrating chloroplast genomic data, nuclear SSR markers, and comprehensive morphological observations, our study provides evidence supporting the conclusion that *D. mutatus* is not a hybrid of *B. grandis* and *B. pervariabilis*, but is a synonym of *D. yunnanicus*.

These findings highlight the critical importance of integrating multiple lines of genomic and morphological evidence to resolve complex taxonomic relationships within bamboo. Our study underscores the value of chloroplast genomes as effective molecular tools for resolving taxonomic ambiguities, contributing to more accurate and effective conservation strategies, germplasm resource management, and future breeding programs in bamboo. In addition, considering the extremely high genetic similarity between *D. mutatus* and *D. yunnanicus*, we recommend that future conservation efforts prioritize the protection of these bamboo populations as a single genetic resource to avoid redundant conservation investments and promote effective management. Furthermore, the clear identification of synonym taxa will facilitate the rational collection, evaluation, and utilization of bamboo germplasm, which can accelerate breeding programs, support genetic diversity studies, and ensure the sustainable use of bamboo resources in forestry and industry.

## Taxonomic treatment

### Dendrocalamus yunnanicus

J. R. Xue & D. Z. Li, Journal of Bamboo Research 1988, 7(4): 1–19.

= *Dendrocalamus × mutatus* T.P.Yi & B.X.Li, Journal of Sichuan Forestry Science and Technology 2015, 36(1): 1–5, syn. nov.

### Type

China. Sichuan Province, Changning County (E104° 56’ 13″, N28° 30’35″), T.P. Yi s.n. (holotypus: SIFS).

### Voucher specimens examined

*Dendrocalamus × mutatus*: Young and healthy leaves were collected from Changning, Sichuan province (28°29′N, 104°58′E); voucher specimen BR09, deposited at the College of Forestry and Biotechnology, Zhejiang A&F University (ZJFC). *Dendrocalamus yunnanicus*: Collected from Jinping, Yunnan Province (22°09′18″N, 103.494°E); voucher specimen BR028, deposited at the Zhejiang A&F University (ZJFC). All samples were identified by Dr. Xinchun Lin (Zhejiang A&F University) and Dr. Chaomao Hui (Southwest Forestry University).

### Distribution

China: Yunnan Province (Jinping), Sichuan Province (Changning). The species occurs both in wild populations and widely cultivated stands.

### References

Hsueh CJ, Li DZ: A study on the genus *Dendrocalamus* Nees from China. *Journal of Bamboo Research* 1988, **7**(4):1–19.

Yi T, Li B, Shi J, Ma l, Yang L: New Taxa of *Dendrocalamus* Nees and Other Species. Journal of Sichuan Forestry Science and Technology 2015, 36(1):1–5.

### Notes

Based on integrated morphological and molecular evidence, *D. mutatus* is here treated as conspecific with *D. yunnanicus* and is regarded as a synonym of the latter.

## Materials and methods

### Plant material and DNA extraction

Young and healthy leaves of *Dendrocalamus* × *mutatus* T.P.Yi & B.X.Li were collected from Changning, Sichuan province (28◦290 N, 104◦580 E). *Dendrocalamus yunnanicus* J. R. Xue & D. Z. Li was collected from Jinping, Yunnan Province (22◦918 N 103.494 E). The voucher specimens are available at the College of Forestry and Biotechnology, Zhejiang A&F University (Accession No. BR09 and BR028). The samples of *D. mutatus* and *D. yunnanicus* were identified by the authors, Dr. Xinchun Lin and Dr. Chaomao Hui, from Zhejiang A&F University and Southwest Forestry University. Additionally, samples of *Bambusa pervariabilis* McClure, *Bambusa grandis* (Q. H. Dai & X. L. Tao) Ohrnb, and *Bambusa pervariabilis* McClure *× Dendrocalamopsis daii* Keng f (*B. pervariabilis × B. grandis*) were collected by Dr. Dayong Huang at the Bamboo Garden of Guangxi Forestry Research Institute, Nanning, Guangxi, China (22°55′37.9572″N, 108°20′50.4384″E). All specimens were identified by local taxonomists, and their voucher samples are preserved at the Guangxi Forestry Research Institute. Fresh leaves of *Dendrocalamus sikkimensis* Gamble ex Oliv were collected from Xishuangbanna Tropical Botanical Garden, Chinese Academy of Sciences, Menglun, Yunnan Province, China (21°55′39″N, 101°15′56″E). The total DNA was extracted using a modified CTAB protocol [69].

### Chloroplast genome assembly and gene annotation

The complete chloroplast genome of *D. mutatus* was sequenced utilizing the Illumina NovaSeq PE150 sequencing platform provided by Novogene Bioinformatics Technology (Beijing, China). After sequencing, we performed quality control to eliminate adapter sequences and low-quality reads, ensuring high-quality clean data for downstream analysis. The chloroplast genome assembly was subsequently accomplished using NOVOPlasty software, optimized specifically for assembling organellar genomes from short reads. Annotation of the assembled genome was conducted by leveraging the GeSeq online platform, supplemented with cross-validation using CPGAVAS2 to enhance annotation accuracy [70–72]. Finally, the visualization of the circular chloroplast genome map was executed using Chloroplot software, providing clear representations of gene distribution, structural regions, and GC content variations [73].

### Microsatellite analysis

To identify repetitive elements within the chloroplast genome of *D. mutatus*, we employed the REPuter tool [74], which was run with its standard configuration to locate long repeat sequences. For the detection of simple sequence repeats (SSRs), we utilized the MISA software [75], specifying the following thresholds: at least ten consecutive bases for mononucleotide repeats, five for dinucleotides, four for trinucleotides, and a minimum of three repeats for each of the tetra-, penta-, and hexanucleotide motifs. In addition, previously developed nuclear SSR markers for bamboo were employed to analyze the nuclear SSR polymorphism among *D. mutatus* and related species [34]. The PCR amplification was carried out in a 10 µl reaction volume containing 5 µl Taq master mix (2x Specific Taq Master Mix, 250 units, novoprotein, Suzhou, China), 2 µl ddH_2_O, 100 ng total DNA and 2 umol/L of each primer. The PCR program followed was initial denaturation of 95 °C for 5 min, followed by 30 cycles of 95 °C for 30 s (denaturation), 55 °C for 30 s (annealing) and 72 °C for 10 s (extension) and a final extension at 72 °C for 10 min. The PCR products were separated in the 3% agarose gels.

### Codon bias usage analysis

We examined codon usage patterns and calculated the relative synonymous codon usage (RSCU) values for all protein-coding genes using the CodonW program [69]. All analyses were performed using the standard parameters provided by the software.

### Comparison of related chloroplast genomes

To investigate the sequence variation among *D. mutatus* and related species, we compared the complete chloroplast genomes of *D. mutatus*, *B. grandis*, *D. sikkimensis*, *B. pervariabilis*, and *D. yunnanicus* using the mVISTA tool [76], which enabled a comprehensive alignment and visualization of genomic differences. In addition, IRscope (https://irscope.shinyapps.io/irapp/) [77] was utilized to examine and graphically present the gene arrangements at the boundaries of the single-copy and inverted repeat regions across eight chloroplast genomes.

### Phylogenetic analysis

Phylogenetic analyses were conducted using 58 Poaceae chloroplast genomes, including all available *Dendrocalamus* species and representative species of *Gigantochloa*, *Phyllostachys*, and *Bambusa*, with *Oryza sativa*, *Avena sativa*, and *Zea mays* as outgroups. The 58 Poaceae species’ complete chloroplast genomes were downloaded from GenBank (Table S3) and aligned using MAFFT v7 software [78]. The optimal nucleotide substitution model was determined by jModelTest v2.1.10, and the JC model was selected for subsequent analyses [79]. Maximum Likelihood (ML) phylogenetic analysis was performed with RAxML-NG [80], using 1000 bootstrap replicates to assess topological robustness. Bayesian Inference (BI) was conducted in MrBayes v3.2.6 [81] with four Markov chains run for two million generations, sampling every 1000 generations. The resulting trees from both ML and BI analyses were combined and visualized with FigTree v1.4.4.

## Supplementary Information

Supplementary Material 1: Table S1. Base composition in the chloroplast genome of *D. mutatus.* Table S2. Long repeats in the *D. mutatus* chloroplast genome. Table S3. List of the chloroplast genome of 58 Poaceae species used for phylogenetic analysis Table S4. Different genefragment clone primer design of *D. mutatus and D. yunnanicus. *Table S5. Details of the primers used in this study.

## Data Availability

The data that support the findings of this study have been deposited in the NCBI database [GenBank accession: PQ369414.1] ([http://www.ncbi.nlm.nih.gov/](http:/www.ncbi.nlm.nih.gov)).
