# Supplementary material for: Chloroplast genome analysis of Dendrocalamus × mutatus and its implications for bamboo classification
Source: BMC Plant Biol. 2025 Sep 1;25:1177. doi: 10.1186/s12870-025-07199-x (PMC12400770; doi:10.1186/s12870-025-07199-x)
Supplement: Supplementary file 1 — Supplementary Material 1: Table S1. Base composition in the chloroplast genome of D. mutatus. Table S2. Long repeats in the D. mutatus chloroplast genome. Table S3. List of the chloroplast genome of 58 Poaceae species used for phylogenetic analysis Table S4. Different genefragment clone primer design of D. mutatus and D. yunnanicus. Table S5. Details of the primers used in this study. [file 12870_2025_7199_MOESM1_ESM.docx]

# Supplementary Material

**Table S1.** Base composition in the chloroplast genome of *D. mutatus*

| Region | Total | A (bp) | T (bp) | C (bp) | G (bp) | A+T (bp) | C+G (bp) | GC content (%) |
| --- | --- | --- | --- | --- | --- | --- | --- | --- |
| LSC | 82,964 | 25,932 | 26,307 | 15,188 | 15537 | 52,239 | 30,725 | 37.00 |
| SSC | 12878 | 4,615 | 3,989 | 2,204 | 2,069 | 8,604 | 4,273 | 33.70 |
| IRb | 21,795 | 6,070 | 6,090 | 4,611 | 5,024 | 12,156 | 9,639 | 44.20 |
| IRa | 21,795 | 6,090 | 6,070 | 5,024 | 4,611 | 12,160 | 9,635 | 44.20 |
| Total | 139,432 | 42,707 | 42,456 | 27,027 | 27,242 | 85,163 | 54,269 | 38.92 |

**Table S2.** Long repeats in the *D. mutatus* chloroplast genome

| No. | Size (bp) | Copy 1 Start | Type | Copy 2 Start |
| --- | --- | --- | --- | --- |
| R1 | 58 | 50501 | F | 50681 |
| R2 | 50 | 126454 | P | 139382 |
| R3 | 41 | 50572 | F | 50656 |
| R4 | 31 | 35843 | F | 106516 |
| R5 | 31 | 19907 | P | 35843 |
| R6 | 30 | 15049 | F | 111375 |
| R7 | 30 | 50464 | F | 50485 |
| R8 | 30 | 90147 | P | 90147 |
| R9 | 30 | 15049 | P | 15049 |
| R10 | 30 | 40071 | P | 40071 |
| R11 | 30 | 89768 | F | 89789 |
| R12 | 30 | 111375 | P | 111375 |
| R13 | 29 | 29648 | P | 68432 |
| R14 | 29 | 50612 | F | 50654 |
| R15 | 28 | 100337 | F | 100355 |
| R16 | 27 | 50572 | F | 50614 |
| R17 | 27 | 34694 | F | 34861 |
| R18 | 26 | 103922 | F | 103967 |
| R19 | 26 | 50560 | F | 50740 |
| R20 | 25 | 706 | F | 125696 |
| R21 | 25 | 125696 | P | 125723 |
| R22 | 25 | 706 | P | 733 |
| R23 | 25 | 733 | F | 125723 |
| R24 | 24 | 130103 | P | 130133 |
| R25 | 24 | 37683 | F | 37751 |
| R26 | 22 | 36486 | F | 113719 |
| R27 | 22 | 12713 | P | 36486 |
| R28 | 21 | 33322 | P | 68435 |
| R29 | 21 | 50845 | R | 50845 |
| R30 | 21 | 29653 | F | 33322 |
| R31 | 21 | 37713 | P | 69653 |
| R32 | 20 | 48498 | F | 48519 |
| R33 | 20 | 50527 | F | 50815 |
| R34 | 20 | 50707 | F | 50815 |
| R35 | 20 | 73613 | R | 73613 |
| R36 | 20 | 94677 | P | 94702 |
| R37 | 20 | 27883 | R | 27883 |
| R38 | 20 | 50522 | F | 50855 |
| R39 | 20 | 50702 | F | 50855 |
| R40 | 20 | 62069 | F | 64293 |
| R41 | 20 | 89737 | F | 89758 |
| R42 | 20 | 127027 | P | 127027 |
| R43 | 19 | 496 | R | 496 |
| R44 | 19 | 7180 | P | 71510 |
| R45 | 19 | 17480 | C | 108955 |
| R46 | 19 | 39621 | R | 39621 |
| R47 | 19 | 496 | C | 125939 |
| R48 | 19 | 17480 | R | 17480 |
| R49 | 19 | 30061 | F | 30078 |

**Note**. F, forward repeats; P, palindromic repeats; C, complement repeats; R, reverse repeats.

**Table S3**. List of the chloroplast genome of 58 Poaceae species used for phylogenetic analysis

| Latin Name | Genus | NCBI Accessio |
| --- | --- | --- |
| *Avena sativa* | *Avena* | MK336398.1 |
| *Bambusa arnhemica* | *Bambusa* | NC_026958.1 |
| *B. albolineata* | *Bambusa* | MW557324.1 |
| *B. bambos* | *Bambusa* | NC_026957.1 |
| *B. basihirsuta* | *Bambusa* | NC_050773.1 |
| *B. bicicatricata* | *Bambusa* | NC_050772.1 |
| *B. boniopsis* | *Bambusa* | NC_050754.1 |
| *B. contracta* | *Bambusa* | NC_059751.1 |
| *B. cornigera* | *Bambusa* | NC_050770.1 |
| *B. dolichoclada* | *Bambusa* | NC_063133.1 |
| *B. dolichomerithalla* | *Bambusa* | NC_063131.1 |
| *B. emeiensis* | *Bambusa* | NC_015830.1 |
| *B. flexuosa* | *Bambusa* | NC_050774.1 |
| *B. lapidea* | *Bambusa* | NC_059750.1 |
| *B. odashimae* | *Bambusa* | NC_050771.1 |
| *B. teres* | *Bambusa* | NC_050751.1 |
| *B. grandis* | *Bambusa* | ON000095.1 |
| *B. stenoaurita* | *Bambusa* | NC_058217.1 |
| *B. variostriata* | *Bambusa* | NC_063090.1 |
| *B. oldhamii* | *Bambusa* | NC_012927.1 |
| *B. pervariabilis* | *Bambusa* | NC_053748.1 |
| *Dendrocalamus barbatus* | *Dendrocalamus* | MK679769.1 |
| *D. brandisii* | *Dendrocalamus* | NC_050763.1 |
| *D. farinosus* | *Dendrocalamus* | OM177223.1 |
| *D. fugongensis* | *Dendrocalamus* | NC_050764.1 |
| *D. hamiltonii* | *Dendrocalamus* | NC_050746.1 |
| *D. latiflorus* | *Dendrocalamus* | NC_013088.1 |
| *D. liboensis* | *Dendrocalamus* | NC_081047.1 |
| *D. membranaceus* | *Dendrocalamus* | NC_050766.1 |
| *D. minor var. amoenus* | *Dendrocalamus* | MK679791.1 |
| *D. mutatus* | *Dendrocalamus* | PQ369414.1 |
| *D. pachystachyus* | *Dendrocalamus* | NC_050753.1 |
| *D. pulverulentus* | *Dendrocalamus* | NC_050758.1 |
| *D. sapidus* | *Dendrocalamus* | NC_050757.1 |
| *D. semiscandens* | *Dendrocalamus* | NC_050748.1 |
| *D. sikkimensis* | *Dendrocalamus* | NC_050760.1 |
| *D. sinicus* | *Dendrocalamus* | NC_045941.1 |
| *D. yunnanicus* | *Dendrocalamus* | NC_050761.1 |
| *D. strictus* | *Dendrocalamus* | NC_050776.1 |
| *D. bambusoides* | *Dendrocalamus* | NC_050762.1 |
| *Gigantochloa parviflora* | *Gigantochloa* | NC_050749.1 |
| *G. albociliata* | *Gigantochloa* | NC_050765.1 |
| *G. glabrata* | *Gigantochloa* | MK679788.1 |
| *G. nigrociliata* | *Gigantochloa* | NC_050778.1 |
| *G. verticillata* | *Gigantochloa* | NC_050779.1 |
| *Oryza sativa* | *Oryza* | NC_031333.1 |
| *Phyllostachys edulis* | *Phyllostachys* | PP545474.1 |
| *P. heteroclada* | *Phyllostachys* | NC_064526.1 |
| *P. angusta* | *Phyllostachys* | NC_053647.1 |
| *P. aureosulcata* | *Phyllostachys* | NC_071867.1 |
| *P. glauca* | *Phyllostachys* | NC_051535.1 |
| *P. incarnata* | *Phyllostachys* | NC_067630.1 |
| *P. lithophila* | *Phyllostachys* | NC_062169.1 |
| *P. makinoi* | *Phyllostachys* | NC_062168.1 |
| *P. sulphurea* | *Phyllostachys* | NC_024669.1 |
| *P. propinqua* | *Phyllostachys* | NC_016699.1 |
| *P. violascens* | *Phyllostachys* | NC_068835.1 |
| *Zea mays* | *Zea* | NC_001666.2 |

**Table S4**. Different gene fragment clone primer design of *D. mutatus and D. yunnanicus*

| Primer-F | AAGGATAGAAAGGCCGTGAGGAC |
| --- | --- |
| Primer-R | GCCATTGCAATTGCCGGAAATAC |
| *D. mutatus* clone gene fragment | AGGATAGAAAGGCCGTGAGGACGGGAAAAGAAAAATCAAATCTTTTGAATTTTTAATTAGTTCTCTTTTTTTGCAATTTTCTTATTATCCATTCCATTCATTTTTTTTTATAGAATACTAAAGTATTCTATAAAAAATCTTTATTTTGCAAACTAAAAAATACAATAGTCAATATTCCTTATAATAGATATACTTAATTATATTATAAGAATCTTAAGATATTTTTCGAATAGATAGAAATAGTAAATTTGAATTGAGACACCTATTCTATGACGGATTTTAACTTACCTTCTATTTTCGTGCCTTTAGTAGGCTTAGTATTTCCGGCAATTGCAATGGC |
| *D. yunnanicus* clone gene fragment | AAGGATAGAAAGGCCGTGAGGACGGGAAAAGAAAAATCAAATCTTTTGAATTTTTAATTAGTTCTCTTTTTTTGCAATTTTCTTATTATCCATTCCATTCATTTTTTTTTATAGAATACTAAAGTATTCTATAAAAAATCTTTATTTTGCAAACTAAAAAATACAATAGTCAATATTCCTTATAATAGATATACTTAATTATATTATAAGAATCTTAAGATATTTTTCGAATAGATAGAAATAGTAAATTTGAATTGAGACACCTATTCTATGACGGATTTTAACTTACCTTCTATTTTCGTGCCTTTAGTAGGCTTAGTATTTCCGGCAATTGCAATGGC |

**Note**. The 2 bp position difference between *D. yunnanicus* and *D. mutatus* was treated with red

Table S5. Details of the primers used in this study

| No. | Primer | Forward primer(5'-3') | Reverse primer(5'-3') | Expected length/bp | Tm/ ℃ | SSR Motif |
| --- | --- | --- | --- | --- | --- | --- |
| 1 | SSR-23 | GTGGACCTGTGGTATGAGAC | AAACTATCCTCGCTGCAATT | 190 | 57 | (A)11 |
| 2 | SSR-24 | CAAGCTCCCACAGAACCTCC | AGACAACACACCGCGCTTAT | 194 | 59 | (AG)8 |
